# Atraumatic Splenic Rupture as the Sole Manifestation of Infectious Mononucleosis: A Case Report and Literature Review

**DOI:** 10.7759/cureus.105885

**Published:** 2026-03-26

**Authors:** Annalisa Coppola, Maria Apostolou, Hugo Dario Delle Donne, Pasquale Ferrante, Pietro Francesco Bagnoli

**Affiliations:** 1 Department of General Surgery, Istituto di Ricovero e Cura a Carattere Scientifico (IRCCS) San Raffaele Scientific Institute, Vita-Salute San Raffaele University, Milan, ITA; 2 Department of General Surgery, Istituto Clinico Città Studi, Milan, ITA; 3 Department of Biomedical, Surgical and Dental Sciences, University of Milan, Milan, ITA

**Keywords:** atraumatic splenic rupture, case report, epstein-barr virus, infectious mononucleosis, splenectomy

## Abstract

Atraumatic splenic rupture is an uncommon but life-threatening complication of Epstein-Barr virus (EBV) infection. While infectious mononucleosis classically presents with fever, pharyngitis, and lymphadenopathy, we recorded a spontaneous splenic rupture even in the absence of these symptoms. We report a case of a 26-year-old male presenting to the emergency department with syncope. A progressive clinical hemodynamic deterioration was detected, ending hemorrhagic shock. Imaging revealed an atraumatic grade V splenic injury and massive hemoperitoneum. Laboratory tests showed lymphocytosis, prompting EBV serology, which confirmed acute mononucleosis infection. Due to hemodynamic instability, the patient underwent splenectomy with uneventful recovery. This case highlights that EBV-related splenic rupture may represent the only manifestation of infection in otherwise asymptomatic individuals. Clinicians should maintain a high index of suspicion for EBV infection in patients presenting with unexplained atraumatic splenic rupture. Early diagnosis and timely management are crucial to reduce the risk of potentially life-threatening complications.

## Introduction

Infectious mononucleosis (IM) represents the most common clinical manifestation of primary Epstein-Barr virus (EBV) infection and is classically characterized by the triad of fever, pharyngodynia, and cervical lymphadenopathy. It is often accompanied by hepatosplenomegaly, rash, and a characteristic peripheral blood picture with lymphocytosis and atypical lymphocytes [1,2]. Even though other viral infections may cause a mononucleosis-like syndrome associated with lymphocytosis and hepatosplenomegaly, EBV infection remains the most common etiology and should be considered first in the diagnostic workup.

Although the classic clinical triad of IM remains the most common presentation of EBV infection, a recent systematic review and meta-analysis demonstrated that the absence of these typical symptoms does not reliably exclude the disease [3]. Splenomegaly is a well-known associated finding during the course of IM, occurring in approximately 50% of patients [4]. While IM is typically a benign and self-limiting disease, splenic rupture occurs in fewer than 0.5% of cases and represents one of its most serious complications. The clinical presentation may vary widely, ranging from vague left upper quadrant abdominal pain to signs of acute hemorrhage, including hypotension, tachycardia, and syncope [5,6]. Non-operative management (NOM) may be considered for hemodynamically stable patients; however, urgent surgical intervention is required for hemodynamically unstable patients. Early diagnosis and appropriate management are crucial to reducing morbidity and mortality [7].

In this report, we describe a case of a young patient who underwent emergency splenectomy for spontaneous splenic rupture as the sole manifestation of IM. We discuss the clinical presentation, diagnostic workup, surgical management, and relevant literature regarding this rare but potentially life-threatening complication. This case highlights the importance of considering EBV infection in the differential diagnosis of patients presenting with spontaneous splenic injury, even in the absence of typical symptoms of infectious mononucleosis.

## Case presentation

This case report was prepared in accordance with the CARE guidelines. A 26-year-old man presented to the emergency department with a history of a syncopal episode occurring approximately 1 h prior to admission. During the triage assessment, the patient presented a blood pressure of 110/80 mmHg, a heart rate of 90 beats per minute (bpm), a peripheral oxygen saturation of 99%, and a Glasgow Coma Scale of 15. According to regional triage guidelines, the patient was assigned a code 2, indicating potential clinical instability despite the absence of other symptoms. The patient was admitted for clinical observation in the emergency department with continuous monitoring of vital signs. A detailed personal, family, medical, and surgical history was obtained, and revealed no significant findings. The patient denied any recent trauma or strenuous physical activity. He reported only progressive fatigue over the preceding three days, culminating in the syncopal episode, which was not associated with trauma as confirmed by witnesses. Physical examination did not reveal any significant pathological findings. Laboratory results at admission are summarized in Table 1.

**Table 1:** Laboratory results of the patient at admission to emergency department.

Test	Result	Reference range
White blood cell count	14.9×10⁹/L	4-11×10⁹/L
Neutrophils	6.3%	40-75%
Lymphocytes	52.5%	20-45%
Hemoglobin	14.2 g/dL	13.0-17.0 g/dL
Hematocrit	43%	36-46%
Platelets	135×10⁹/L	150-400×10⁹/L
Creatinine	1.05 mg/dL	0.6-1.2 mg/dL
C-reactive protein	15.4 mg/L	<5 mg/L

Approximately 1 hour later, the patient developed sudden hemodynamic deterioration with tachycardia (120 bpm), hypotension (60/40 mmHg), and severe pain in the left upper abdominal quadrant radiating to the chest and left shoulder. Arterial blood gas analysis revealed a hemoglobin level of 6.3 g/dL.

**Figure 1:**
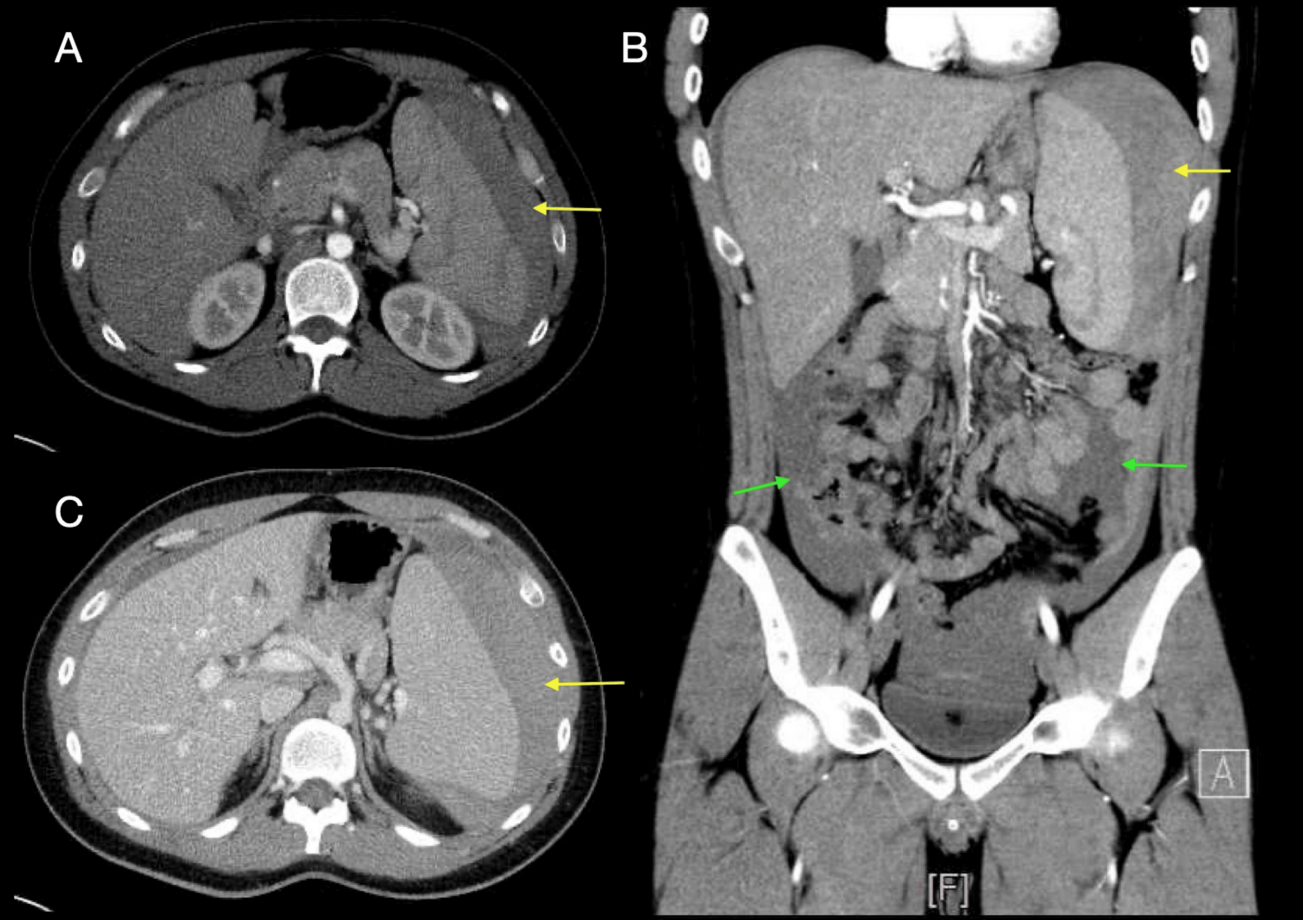
Computed tomography of the patient showing the splenomegaly and splenic rupture, perisplenic hematoma, and hemoperitoneum. (A) Axial arterial-phase image, (B) sagittal arterial-phase reconstruction, (C) axial venous-phase image. The yellow arrows indicate a perisplenic hematoma, and the green arrows indicate free intraperitoneal fluid, hemoperitoneum.

A bedside focused assessment with sonography in trauma (FAST) ultrasound examination demonstrated free fluid in the perisplenic space and in the Douglas pouch, associated with a subcapsular hematoma and splenomegaly. The patient developed hemorrhagic shock and required immediate fluid resuscitation with 500 mL of crystalloids and blood transfusion (2 units of packed red blood cells), which resulted in temporary hemodynamic stabilization. A splenic injury associated with splenomegaly was suspected, and a contrast-enhanced abdominal CT scan was performed (Figures 1A-1C). The scan revealed hemoperitoneum, splenomegaly (16 cm in diameter), a perisplenic hematoma, and a grade V splenic injury according to the American Association for the Surgery of Trauma (AAST) classification, characterized by complete fragmentation of the splenic parenchyma [8]. The coexistence of lymphocytosis, splenomegaly, and splenic rupture, even in the absence of trauma or the classic clinical triad of infectious mononucleosis, prompted suspicion of EBV infection. Serological testing revealed positive viral capsid antigen (VCA) IgM antibodies, confirming acute EBV infection [9].

Due to the patient’s hemodynamic instability and the radiological findings, operative management was performed. A midline open laparotomy was performed, revealing hemoperitoneum with approximately 1.5 L of intraperitoneal blood. Exploration of the abdominal cavity showed a markedly enlarged spleen (15-18 cm) and a large perisplenic hematoma, with no abnormalities in the liver, pancreas, colon, and small intestine. A splenectomy was performed, which led to the patient's rapid hemodynamic stability, and the total operative time was 45 min. The sample was sent for histopathological examination. Intraoperatively, hemodynamic stability was further supported by administration of an additional 2 units of packed red blood cells.

The postoperative course was uneventful, with progressive improvement and stabilization of hemoglobin levels following multiple blood transfusions. The patient was discharged on postoperative day seven in good general condition, with no complications reported during follow-up. Pneumococcal, meningococcal, and *Haemophilus influenzae* type B vaccinations were scheduled two weeks after splenectomy according to the World Society of Emergency Surgery (WSES) recommendations [10].

Histopathological examination revealed a spleen measuring 13×11.7×5 cm with a partially disrupted capsule in multiple areas. On the cut section, the splenic parenchyma showed marked congestion of the red pulp with subcapsular hemorrhagic extravasation. Expansion of the white pulp was also observed, with lymphoid follicles displaying prominent pale germinal centers. These findings were consistent with acute EBV-related reactive lymphoid hyperplasia and vascular congestion, supporting the diagnosis of atraumatic splenic rupture secondary to acute EBV infection.

## Discussion

Infectious mononucleosis caused by EBV is usually a self-limiting disease that resolves over several weeks to months without long-term sequelae. Although the classical triad of fever, pharyngodynia, and lymphadenopathy is commonly present [11], a recent systematic review and meta-analysis by Cai et al. demonstrated that the absence of these typical symptoms does not reliably exclude the disease [3]. Spontaneous splenic rupture in patients with EBV infection, as reported in our case, can occur even in the absence of prodromal infectious mononucleosis (IM) symptoms and may lead to rapid clinical deterioration, posing a potentially life-threatening risk if not promptly diagnosed. Atraumatic splenic ruptures (ASRs) are rare, with an overall incidence of approximately 0.5% and a reported mortality rate of 12% [12]. An ASR associated with EBV infection is therefore considered extremely uncommon.

The most comprehensive recent systematic review by Toti et al. identified 186 cases of splenic rupture associated with EBV infection between 1970 and 2022; however, this review did not differentiate between traumatic and atraumatic ruptures [13]. Another systematic review by Bartlett et al. reported only 72 cases of ASR in IM, without describing other infection-related symptoms [14]. This case represents a rare presentation of EBV infection, in which atraumatic splenic rupture occurs as the sole initial manifestation, without prodromal symptoms of infectious mononucleosis. Due to the absence of prospective studies, the true incidence of EBV-related silent spontaneous splenic rupture remains unknown.

The pathophysiological mechanism is based on the capacity of the EBV virus to induce a strong immune response primarily targeting infected B lymphocytes, with marked CD8+ T-cell expansion. This process leds to lymphoid hyperplasia within the white pulp and severe vascular congestion in the red pulp, resulting in progressive splenomegaly and increased splenic parenchymal fragility [15]. Splenomegaly is observed in approximately 44% of symptomatic adult patients with IM [16]. Progressive thinning and stretching of the splenic capsule, associated with architectural disruption of the trabecular support system by atypical lymphocyte infiltration, predispose the spleen to rupture even in the absence of trauma [17]. Minimal internal stressors, such as coughing, sneezing, or defecation, may be sufficient to trigger capsular disruption. ASR predominantly affects males (60-70%) and occurs most frequently in adolescents and young adults aged 15-30 years, typically within the first three weeks after the onset of mononucleosis symptoms [14,18].

The differential diagnosis of ASR can be challenging. In patients presenting with syncope and sudden hemodynamic instability, ASR should be considered. Ultrasound (US) is often the first-line imaging modality due to its rapidity and bedside availability [19], whereas contrast-enhanced computed tomography (CT) remains the diagnostic gold standard, providing AAST grading that guides management decisions in hemodynamically stable versus unstable patients [20].

Management of ASR in IM is similar to that of traumatic splenic rupture. According to Toti et al., splenectomy was performed in 58% of patients with EBV-related splenic rupture, non-operative management was attempted in 34%, and 7.5% of patients initially managed non-operatively ultimately required splenectomy [13]. Current WSES guidelines recommend splenectomy for hemodynamically unstable patients, those with high-grade splenic injuries, ongoing hemorrhage, or in settings where embolization is unavailable due to limited resources [10]. In the present case, the patient presented with a high-grade (AAST V) splenic injury, massive hemoperitoneum, and rapid-onset hemorrhagic shock, necessitating urgent surgical management.

## Conclusions

ASR is a rare but potentially life-threatening complication of EBV infection. Although most cases present with the classic symptoms of infectious mononucleosis, spontaneous splenic rupture can, in rare instances, occur as the sole initial manifestation of EBV infection, as demonstrated in the present case. Only a limited number of similar cases have been described in the literature. This report highlights the importance of maintaining a high index of suspicion for EBV-related ASR in young adults presenting with unexplained hemoperitoneum or shock, even in the absence of classic symptoms. Rapid imaging assessment and timely, guideline-driven intervention, in accordance with WSES recommendations, are essential to reduce morbidity and mortality in this potentially fatal condition.
